# Our Bureau of Information

**Published:** 1912-03-02

**Authors:** 


### OUR BUREAU OF INFORMATION.

### Are Guardians Short-Sighted?

It is such a common thing to abuse Boards of Guardians —so easy to gibe at them as being hard-hearted and indifferent to the poor—that one hesitates to add a voice to the general clamour. Some Boards, indeed, while sanatoria for consumption were still in the air and had not materialised on earth, were far-sighted enough to send tuberculous cases to country or seaside homes, where they could have something approaching an open-air treatment. Although they had no legal powers, they did their best to persuade feeble-minded girls who had one child to remain in homes where they could be protected. They realised that though to do these things meant immediate cost to the union, this was less than the ultimate expense of maintaining an entire family. The parish doctor is, indeed, secured for the poor, and the Poor Law infirmary is a boon which is not as much appreciated as it ought to be; but there are extensions of relief which are still very rare and would be valuable.

Let us instance a case which was brought before us. A boy had disease of the middle ear, and was attended by the parish doctor, who said the child ought to be sent to some place in the country where he could have good food and be under the charge of a trained nurse. Given this treatment for a year or two the disease would probably be conquered. Without it the child would become deaf. The child's mother was willing to contribute something to his maintenance, so it should not have been difficult, if the Guardians had been ready to add the ordinary boarding-out allowance, to find a suitable home for him. But they would not do so. Private charity stepped in and met the need, as, happily, private charity so often does in our country. But a man who is incurably deaf is much more likely to become a pauper than a man who has all his senses. Guardians do not grudge the weekly dole to sustain life, but they grudge the larger expenditure at the moment, which would in all likelihood be an investment in health.

### Answers to Correspondents.

Isolation in Diphtheria.

Miss J. D. writes: I am anxious about a little boy who has been suffering from diphtheria for thirteen weeks and is not yet convalescent. All the information we can get from the hospital authorities is that, although quite well in himself, each time a swab is taken from his throat it contains some diphtheria bacillus.

[The boy is convalescent; indeed, he may practically be well. There is no need for anxiety on his account. But he is not entirely free from the bacillus of diphtheria, as the swabs show, and this means that, though he has recovered from the disease himself, he might still be a vehicle of infection to others. It is only right that the authorities should not permit him to leave the hospital until all such danger is past. The long period of isolation necessary in infectious diseases after the patient feels quite well is very trying, both to patient and friends, but in the interests of public health it is absolutely necessary.]

The Youngest Age for a Probationer.

"Anxious One" writes: Could you give me the youngest age that a probationer will be received into a children's hospital, and, if possible, the names of some of the best of these hospitals?

[See reply to "Ferndale." It would be invidious to name one among the many good children's hospitals in the country. If you do not want to go very far from your home, the children's hospital at Pendlebury might suit you. You will find all information on the subject in the "Hospitals and Charities Annual," edited by Sir Henry Burdett, which you will probably find in your public library. Or you can obtain it from the Scientific Press.]

Home for Chronic Invalids.

"C. M. B." writes: I am a mental nurse. Could you give me the addresses of Homes where chronic invalids are received in Hornsey?

Medical Electricians.

"Electric" writes: Can you give me any particulars, such as qualifications, salary, etc., and any other information that will enable me to become a medical electrician; also where I could obtain the necessary training? I am a young electrician, and would like to take up this branch of the profession, but do not know where to obtain the necessary training, nor what are the prospects of the work.

["M.R.C.S." answers: It is generally recognised that the medical employment of electricity in medicine should be taken up only by medical men. If your correspondent is a registered medical man he should write to the Dean of the Medical School of Guy's Hospital, which is, I understand, regarded as the best school for this training.]

### Our List of Approved Homes.

We have pleasure in adding the following to our list of Approved Homes:—

"The Orchard," Haslemere. Principals, Sister Herbert and Miss Maurice. Convalescent home for ladies and children. Surgical and nerve cases needing special care received. House situated 600 feet above sea-level, in the neighbourhood of pinewoods. Terms: Ladies from three guineas a week; children from two guineas a week.

4 Magdalen Road, St. Leonards-on-Sea. Principal, Miss Moody. Medical, surgical, and convalescent cases. Good operating theatre. Permanent cases received. Terms from three guineas per week.

### Rules for Correspondents.

Everyone who uses or wishes to help our Bureau of Information must be kind enough to observe the following rules:—

(1) Postal replies cannot be given. Exception will only be made in very special cases at the Editor's discretion.

(2) Queries or answers relating to different cases must be written, or preferably typed, on separate sheets of paper, and the writing must never be crossed.

(3) Questions and replies received not later than Wednesday will, as far as possible, be dealt with in our issue of the following Saturday week.

(4) Letters must have attached the name and address of the sender, with a pseudonym for publication if preferred.

(5) Applications referring to non-dependents where the need relates to business or employment must be accompanied by a postal order for 5s., when the requirement shall have publicity in these columns.

(6) All applicants for insertion in our list of Approved Homes must send a registration fee of five shillings. The omission of this leads to delay and gives avoidable trouble.

(7) *All communications* for this department *must be accompanied by the coupon* to be found at the bottom of the third page of the cover on the inside, and should be addressed to the Editor of the Bureau of Information, c/o The Hospital, 29 Southampton Street, Strand, London, W.C.

